# Predictors of Atrial Fibrillation Recurrences after a First Radiofrequency Catheter Ablation Intervention for Paroxysmal Atrial Fibrillation—Experience of a Low Volume Ablation Centre

**DOI:** 10.3390/medicina57111139

**Published:** 2021-10-20

**Authors:** Lavinia-Lucia Matei, Călin Siliște, Sebastian Stoica, Gabriel-Cristian Bejan, Liviu-Nicolae Ghilencea, Dragoș Vinereanu

**Affiliations:** 1 Cardiothoracic Pathology Department, University of Medicine and Pharmacy Carol Davila, 020021 Bucharest, Romania; amarielavinia@yahoo.com (L.-L.M.); calin_siliste@yahoo.com (C.S.); cristian.bejan@umfcd.ro (G.-C.B.); 2 Department of Cardiology, Elias Emergency University Hospital, 011461 Bucharest, Romania; 3 Department of Cardiology and Cardiovascular Surgery, University and Emergency Hospital, 050098 Bucharest, Romania; sebastian.stoica66@gmail.com

**Keywords:** atrial fibrillation, ablation, atrial fibrillation recurrence, continuous ECG monitoring

## Abstract

*Background and Objectives:* Atrial fibrillation recurrences (AFR) after radiofrequency catheter ablation (RFCA) are not uncommon, up to 65% of patients having relapses in the first year. However, current data are based mainly on studies from centres with a large volume of ablations, as they include technically inhomogeneous interventions, and populations with different types of AF. The aim of our study was to assess and stratify the risk at 6 and 12 months for AFR after a single RFCA, in patients with paroxysmal AF, in a centre with low volume activity. *Materials and Methods:* We enrolled 40 patients who underwent an initial RFCA, followed by continuous 48 h ECG monitoring at 1, 3, 6, and 12 months. Patients self-monitored their cardiac activity by random daily radial pulse palpation or in the presence of palpitations. *Results:* Ten independent predictors for late AFR were identified, and a 6-month risk score was computed using three of them: AFR duration in the first month, number of AFR between 1 and 3 months, and supraventricular ectopics per 24 h at 6 months. The score can explain 59% of the AFR (*p* = 0.001). A further 12-month assessment identified three independent predictors. The presence of AFR between 6–12 months is the most important of them (OR = 23.11, 95% CI = 3.87–137.83, *p* = 0.001), explaining 45% of AFR over 1 year. The risk scores at 6 and 12 months were internally validated. *Conclusions:* The 6-month score proved to be a useful tool in guiding further strategy for patients with a low risk, while a longer follow-up to 12 months may avoid unnecessary early reinterventions.

## 1. Introduction

Catheter ablation for atrial fibrillation (AF) is a feasible treatment strategy for patients with symptomatic drug-refractory AF [1,2,3,4,5]. It reduces the arrhythmic burden, while haemodynamic parameters and quality of life improve [6,7,8,9,10,11]. The intervention has favourable outcomes [12,13], but almost half of patients will have recurrences in the first year [1,8,14,15]. Early recurrences are frequently encountered [16], but do not always predict an unfavourable outcome [17,18,19]. After the first three months, atrial fibrillation recurrences (AFR) are associated with a higher recurrence risk in long-term monitoring [17,20,21].

Decision and timing for reintervention are guided by both electrical and morphological characteristics and depend on the patient’s symptoms. Risk prediction scores have been designed; however, most of them rely on multiple parameters (clinical, electrical, echocardiography measurements, and type of intervention), are derived from inhomogeneous populations, and imply periodically in-hospital evaluations [22]. Atrial ectopics are triggers for AF episodes [1,18] and an increased number of supraventricular premature beats (SVPB) [23,24,25,26,27] correlates with a high risk of AF recurrences after ablation.

The aim of this study was to identify predictors for AFR and to develop a risk score that might help to predict the outcome, as well as the optimal time for a second intervention.

## 2. Methods

### 2.1. Study Design

We consecutively enrolled 40 patients who underwent an initial RFCA intervention for symptomatic drug resistant paroxysmal AF. Inclusion criteria were a history of episodes of paroxysmal AF and normal left ventricular ejection fraction (LVEF > 50%). Exclusion criteria referred to nonparoxysmal AF episodes, repeat radiofrequency catheter ablation (RFCA), ischemic or structural myocardiopathies, significant valvular diseases, and uncontrolled risk factors for AFR (thyroid disease, sleep apnoea, alcohol consumption). All patients signed the informed consent, and the study protocol was approved by the Local Research Ethics Committee.

Briefly, our ablation protocol is described as follows: a decapolar catheter with 5 mm electrodes and 2 mm interelectrode spacing is placed in the coronary sinus either by superior or inferior approach. The left atrium access is through 2 transseptal punctures, performed under contrast and pressure control. Two catheters are placed in the left atrium: a circular duodecapolar catheter with 2-6-2 interelectrode distance (Lasso 2151 Biosense Webster, Inc., Diamond Bar, CA, USA) and a 3.5 mm tip ablation unidirectional catheter (Thermocool Smartouch Biosense Webster, Diamond Bar, CA, USA). Before transseptal puncture, heparin is initiated to achieve a target activated clotting time of 300–350 s, monitored at each 30 min; additional boluses are added if necessary.

All patients underwent preprocedural CT examination of the left atrium (LA) and transesophageal echocardiography. Three-dimensional electroanatomic mapping is performed with a CARTO (Biosense Webster, Inc., Diamond Bar, CA, USA) system. All bipolar electrograms are filtered at 30 to 150 Hz and displayed on a commercially available electrophysiological recording system (Cardiolab, GE, Houston, TX, USA). The ablation lines are placed at the proximal pulmonary vein antrum as suggested by the LA CT reconstruction. Isolation of all the PVs (regardless of the presence or absence of spontaneous local activity) is performed by creating circumferential lines (for ipsilateral veins) and with carina ablation if needed. Radiofrequency energy is delivered in the power control mode, with a of limit of 30 W (25 W for posterior wall). Lesions are delivered for a maximum of 60 s. Successful PV isolation is defined by demonstrating entrance and exit block (recording and stimulating the circular catheter). The block is demonstrated using the circulatory catheter or the ablation catheter. No applications are made to the posterior wall when the vein is electrically insulated and the CartoSeg module indicates the proximity of the esophagus. We do not use stimulation protocols to induce AF after the demonstration of block.

We defined AFR as any episode of atrial fibrillation that lasted more than 30 s: very early AFR in the first 3 months (blanking period), early AFR between 3 and 6 months, late AFR between 6 and 12 months, and very late AFR after 12 months. Patients were monitored during hospitalisation with telemetry for the first 5 days. Clinical evaluation and continuous ECG monitoring for 48 h was performed with a GE SEER 1000, 3-channel Digital Holter at 1, 3, 6, and 12 months after RFCA; CardioDay, GE, USA software was used for analysis. Every recording was manually reviewed by an experienced cardiologist. Meanwhile, patients were asked to self-monitor their cardiac activity by daily radial pulse palpation, for at least 1 to 3 min, and additionally in the presence of palpitations. Presence and duration of arrhythmias (cardiac frequency or rhythm irregularities) were reported, and specific measures were recommended if patients described symptoms (palpitations, dyspnea, fatigability that impaired daily activity) or rhythm abnormalities during periods of self-monitoring (urgent ECG or extended ambulatory ECG monitoring).

### 2.2. Statistical Analysis

Continuous variables are presented as mean ± SD (standard deviation) for uniform distribution, and Student’s t-test was used for comparisons of the central tendency of baseline characteristics. Abnormally distributed continuous variables are reported as medians (IQR, interquartile range), and nonparametric tests (Mann–Whitney U rank-sum test or Wilcoxon rank-difference test) were used for comparison. Categorical data are reported as numbers (percentages) and were compared by chi-square test.

We have used G*Power software to assess the power of the statistical method applied to our dataset, with α = 0.05 and power (1–β) = 0.95 (G*Power for Windows 3.1.9.7) [28,29].

Logistic regression was used to extrapolate the results to general population, to better visualise the differences in the number of events between groups, and also to compute a score risk, by combining several covariates in one model wherever possible. The design of our study did not include the time-to-event analysis to make it proper for Cox proportional hazard risk model analysis.

Variables with statistically significant influence over the outcome have been processed with univariate linear regression to identify predictors for AFR (area under the ROC curve, AUROC > 0.650 and Hosmer–Lemeshow goodness-of-fit test *p* > 0.05 and coefficient *p*-value < 0.1). The odds ratio (OR) was generated for each of the identified predictors.

Predictors previously identified have been computed with multivariate logistic regression and the best model was selected based on discrimination (with AUROC curve) and calibration (Akaike’s Information Criterion, AIC and Bayesian Information Criterion, BIC) at the lowest values and Nagelkerke R^2^ test. A threshold for AFR risk was identified in the training cohort and the sensitivity and specificity identified were reported.

Results were validated internally after a random selection of a contingent of patients from the initial cohort [30]. The comparison between the study cohort and validation contingent was calculated using the Hanley and McNeal test.

All *p*-values were two-sided and a *p*-value < 0.05 was considered statistically significant. Data were analysed using SPSS (Statistical Package for the Social Sciences) version 26 software (IBM SPSS Statistics, Armonk, NY, USA; IBM Corp.).

## 3. Results

### 3.1. Patient Characteristics

The study population comprised of 40 patients with a mean age of 56 ± 10 years, 29 males (73%), who underwent a first RFCA for paroxysmal AF. The median duration of follow-up per patient was 2 years. Baseline characteristics of the study population are summarised in Table 1.

**Figure 1:**
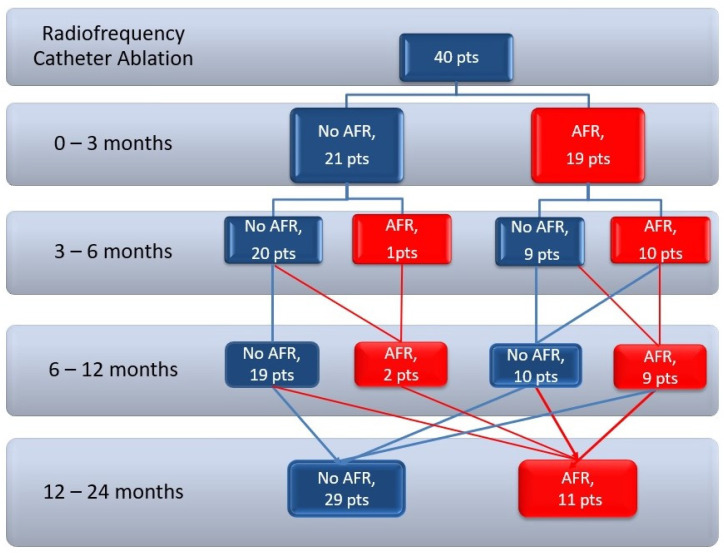
Patients flow chart—outcome after AF ablation. Pts = patients; AF = atrial fibrillation; AFR = atrial fibrillation recurrence.

Atrial fibrillation recurrences during the follow-up period are depicted in Figure 1. Patients reported that most episodes of AFR were symptomatic and self-monitoring detected more AFR episodes than continuous ECG monitoring (Table 2). Atrial fibrillation recurrences diagnosed by self-monitoring correlate well with AF episodes on 48-h ECG Holters (between 0–3 months: r = 0.329, *p* = 0.03; between 3–6 months r = 0.576, *p* < 0.001, between 6–12 months r = 0.610, *p* < 0.001).

Atrial fibrillation recurrences in the blanking period (0–3 months) were strongly correlated with AFR in the first year (r = 0.608, *p* = 0.001), while recurrences after the first year correlated with the presence (r = 0.585, *p* = 0.001) and number of AFR episodes (r = 0.568, *p* = 0.001) recorded between 6 and 12 months (Table A1). We compared different characteristics between patients with AFR and patients without AFR after RFCA, to identify predictors for recurrences (Table 3 and Table 4).

### 3.2. Independent Predictors for AFR between 6 and 12 Months

Patients with AFR between 6–12 months had a significantly higher rate of AFR compared with AFR-free patients during previous periods (between 0–1 months *p* = 0.004, 1–3 months *p* = 0.01, and 3–6 months *p* = 0.002) and also presented more SVPB/24 h at 3 and 6 months Holter ECGs (*p* = 0.005, *p* = 0.009). All statistically significant variables between the groups, measured up to 6 months (Table 3), were evaluated by univariate analysis. Ten independent predictors for late AFR were found: AFR presence, number, and duration of episodes in the first month; AFR presence, number, and duration of episodes between 1 and 3 months; presence and number of AFR between 3 and 6 months, and SVPB by continuous ECG monitoring at 3 and 6 months (Table A2).

**Figure 2:**
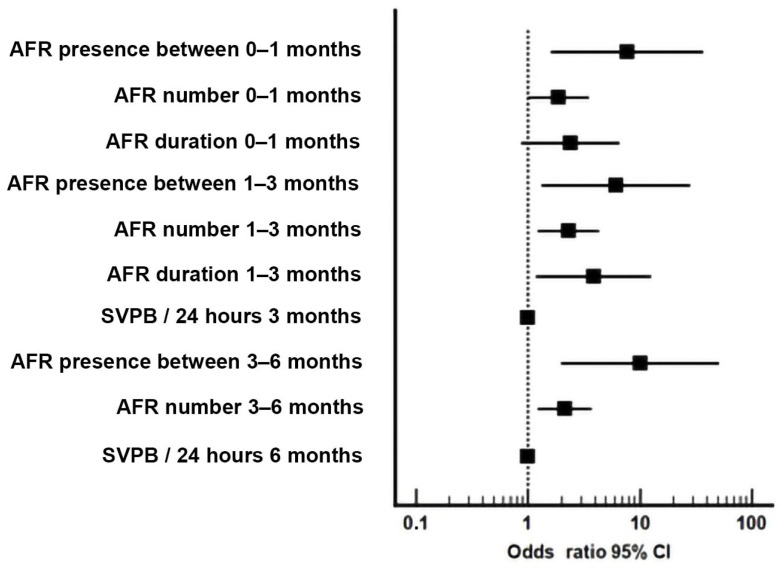
Quantification (Odds Ratio) for AFR prediction between 6 and 12 months after RFCA.

The odds ratio (OR) of the selected characteristics for AFR at six months are detailed in Figure 2.

All found predictors of outcome were analysed using binary logistic regression in order to develop a risk score (Table A3). The best model for predicting AFR between 6 and 12 months comprised of three variables: duration of AFR in the first month, number of AFR between 1 and 3 months, and number of SVPB per 24 h at 6 months of continuous ECG monitoring.

The risk score was computed using the following equation (Table A4):

Six-month risk score = Log (OR AFR 6–12 months) = −6.05 + 2.255 × arctan (Duration of AFR in the first month) + 2.081 × arctan (Number of AFR 1–3 months) + 0.752 × loge (Number of SVPB/24 h at 6 months on continuous ECG monitoring).

The 6-month score explains 59.1% of the late AFR (*p* = 0.001). We determined a threshold of −0.474 (sensitivity 91%, specificity 85%). The Odds Ratio of the 6-month score > −0.474 is 55 (*p* = 0.001), which means that patients with scores over this value have a 55-fold risk of AFR between 6 and 12 months, in comparison with patients with scores less than the cutoff value.

**Figure 3:**
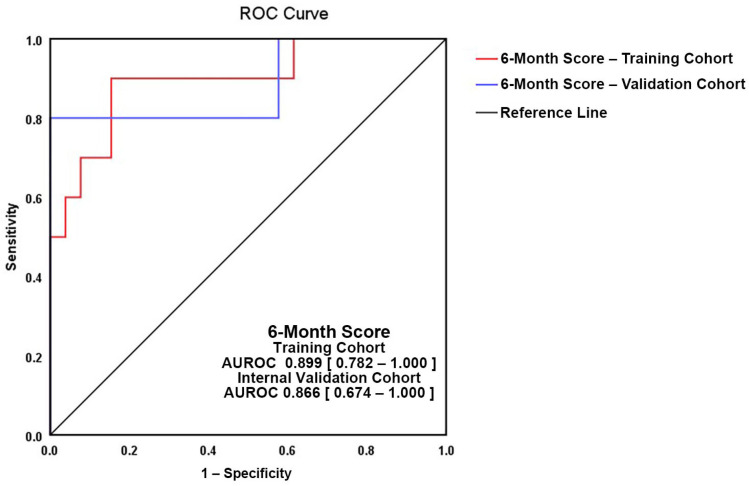
Validation of 6-month Score > −0.474 for AFR prediction between 6 and 12 months after radiofrequency catheter ablation. Red line—study group, blue line—validation subgroup.

Validation of the score (Figure 3) was performed on a randomly assigned subgroup from the study population. The two ROC curves showed good superposition of the training cohort (AUROC = 0.899, 95%CI = 0.782–1.000, *p* = 0.001) and the validation group (AUROC = 0.886, 95%CI = 0.674–1.000, *p* = 0.012) (Figure 3), with a non-significant difference between curve areas of 0.0115 (95%CI = −0.174–0.197, *p* = 0.903).

The power effect of the calculated risk score >−0.474 at 6 months from our cohort is reassuringly higher than the power effect calculated with G*Power software for a sample size of 40 patients (55 versus 6.2, α = 0.05, 1-β = 0.95), which proves that the analysed dataset is big enough for an accurate outcome.

### 3.3. Independent Predictors for AFR after 12 Months

**Figure 4:**
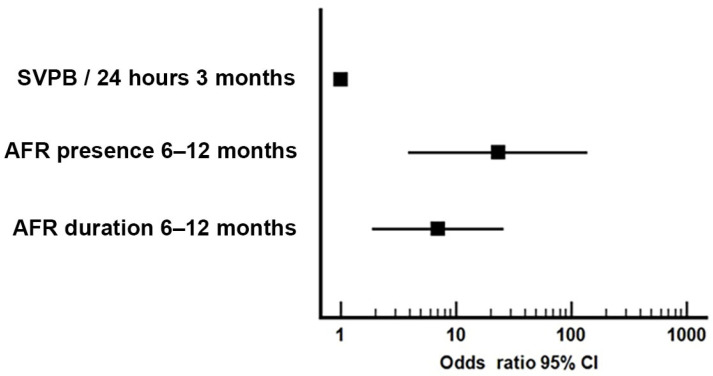
Quantification (Odds Ratio) for AFR prediction after 12 months.

Patients with AFR after 12 months had a significantly higher rate of AFR compared with AFR-free patients only between 6 and 12 months (*p* = 0.001), and a higher number of SVPB/24 h at 6 months and 12 months of ECG Holter monitoring (*p* = 0.023, *p* = 0.001)—Table 4. Three independent predictors for long-term AFR (after 12 months—Table A5) were determined: AFR presence and duration between 6 and 12 months, and number of SVPB per 24 h at 3 months of continuous ECG monitoring (Table A5). Each predictor’s importance was quantified in Figure 4.

AFR between 6 and 12 months qualified as the most important predictor for AF recurrences after the first year. Patients with a history of AFR between 6 and 12 months have a 23-fold higher risk of having long-term AF recurrences (OR = 23.11, 95%CI = 3.87–137.83, *p* = 0.001), and can explain 44.9% of AFR episodes after 12 months (*p* = 0.001)—Table A6.

The regression equation for AFR risk after 12 months was calculated according to the following formula: Log OR (very late AFR) = −2.159 + 3.14 × (AFR 6–12 months).

**Figure 5:**
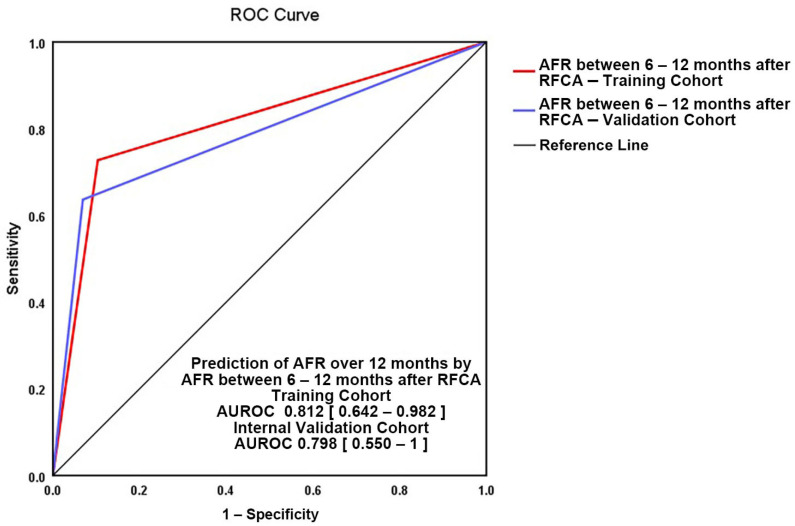
Validation of “AFR between 6–12 months” for atrial fibrillation recurrence (AFR) prediction after 12 months from RFCA. Red line—study group, blue line—validation subgroup.

The validation of our 12-month score model (depicted in Figure 5) was performed internally. The two AUROC curves show good superposition (training cohort: AUROC = 0.812, 95%CI = 0.642–0.982, *p* = 0.001 versus validation cohort AUROC = 0.798, 95%CI = 0.550–1, *p* = 0.039) with no significant difference between areas (0.028, 95%CI = −0.179–0.235) and a Hanley and Mc Neil test *p*-value of 0.789.

The power effect of the predictor at 12 months from our cohort is reassuringly higher than the power effect calculated with G*Power software for a sample size of 40 patients (23.11 versus 6.2, α = 0.05, 1 − β = 0.95) which proves that the analysed dataset is big enough for an accurate outcome.

## 4. Discussion

### 4.1. Rationale for the Study

Radiofrequency catheter ablation for AF is an approachable technique for treating AF, crucial in reducing morbidity and associated healthcare costs. Risk prediction at 6 and 12 months may prove feasible in decision strategy guiding and time to reintervention.

There are limited data addressing AF recurrence risk after the first RFCA in selected patients with paroxysmal AF, normal ejection fraction, and without severe associated comorbidities. We based our data on the experience of a low volume ablation centre, which is an advantage due to the homogenous ablation technique and distinctive statistical design of the study. Our study suggests a two-step strategy based on a computed 6-month risk score and 12-month predictors that can be easily self-monitored. It may prove an important instrument in predicting response after AF RFCA, to determine the optimal timing for reintervention.

### 4.2. Added Value to Current Literature

Albeit our study is based on a limited number of patients, we consider the research valuable due to the statistical methods applied. The risk stratification at 6 and 12 months show a good power of prediction (59.1 and 44.9%, respectively).

Our results support the idea that an intermediate assessment at 6 months after RFCA proves to be beneficial in a two-step strategy with a final assessment at 12 months.

The statistical analysis confirmed the observations that the patients with recurrences in the first 6 months after AF ablation may be AF-free in the following 6 months. The 6-month assessment with the risk score based on three predictors allows us to predict further AF recurrences up to one year. Although the predictors of the score: duration of AFR in the first month, number of AFR between 1 and 3 months, and number of SVPB per 24 h at 6-month continuous ECG monitoring, can explain a small amount of AFR in the following 6 months, the 6-month score explains much more than each predictor separately (59.1% versus 11.4, 29.8, and 48%, respectively).

A second assessment at 12 months found one predictor for AF recurrences: AFR between 6–12 months, which explains 44.9% of AF episodes after the first year.

In brief, the 6-month score explains AF recurrences between 6 and 12 months, while the AF recurrence between 6 and 12 months itself is the only predictor for AF recurrences after one year at the 12-month assessment.

The two-step strategy is a result of a research in a low volume centre. The small analysed dataset is not an obstacle as the inclusion and exclusion criteria of the study were strictly achieved, as we excluded from the dataset all patients with non-paroxysmal AF.

Although the study cohort is small (40 patients), the power effect for both 6-month and 1-year assessment is far more significant than that needed for a sample size similar to our dataset (55 versus 6.2 and 23.11 vs. 6.2, respectively).

### 4.3. Comparison with Published Data

Atrial fibrillation recurrences are frequent after RFCA and arrhythmia monitoring is essential to assess the outcome, in order to influence decisions regarding treatment or reintervention. Repeated ablations are recommended depending on symptoms, although recurrences after the intervention are usually less symptomatic.

Most of the studies found age, gender, comorbidities, non-paroxysmal AF, or type of intervention [22,31,32,33] as independent predictors for AFR. We found no correlations between characteristics of patients, presence of cardiovascular risk factors, and risk of AFR. A possible explanation may be the homogenous population, only with history of paroxysmal AF, normal ejection fraction, and lack of severe comorbidities. The ablation technique also followed the same protocol and was performed according to the latest recommendations.

In our cohort, after excluding the blanking period, 72.5% of patients remained AF free at 1 year, after a single RFCA procedure, similar to published data. Thus, 53–80% of patients will remain AF free after RFCA interventions [34]. Our study demonstrates the clinical applicability of self-monitoring as part of follow-up protocol after AF RFCA, since two thirds of patients had AFR diagnosed by self-monitoring, whereas only one third had AFR episodes recorded on continuous ECG monitoring. The high percentage of AFR diagnosed by self-monitoring may be explained by the presence of more symptomatic episodes corroborated with increased awareness, and patients being trained to monitor their heart rate and rhythm on a daily basis.

Symptomatic supraventricular beats predict AF [24,25,27]. Usually, SVPB burden decreases after a successful ablation, and if atrial ectopy increases over time, AFR may be suspected [20,21,35]. A subanalysis from the MANTRA-PAF study determined that more than 213 SVPB per day after catheter ablation implies a 3-fold higher risk of late AFR, [21] while Gang et al. study showed that more than 142 SVPB/day correlate to late AFR [20]. We also found that a higher number of SVPB were recorded on continuous ECG monitoring in patients who developed latter AFR, by comparison with patients AF-free.

After RFCA different periods are defined, as electrical and morphological remodelling continues, and different mechanisms are described: early recurrences after AF ablation are defined as arrhythmias in the first 3 months after the intervention, while late recurrences are known to appear between 3 and 12 months after the ablation [34]. Recent studies show that the period between 3 and 12 months should also be divided, since AFR that occur between 6 and 12 months have different characteristics and imply a different outcome [36].

The first 3 months after RFCA are known as a “benign or blanking period”, with inflammatory changes and lesions of consolidations after RFCA, assumed as different causes of AFR. Recurrences are common and usually considered not relevant for long-term outcome: up to 50% of patients present AFR, and only half will develop later AFR [34]; on the other hand, absence of AFR predicts a successful outcome in the first year [37] and is associated with better long-term success rates [17,18].

Regarding early assessment, recent studies show that AFR, particularly in the latter part of the blanking period, after the first month, can predict future AFR [18,37,38]. Willems et al. showed that patients with AFR in the second part of the blanking period also have higher risk of long-term AFR (more than 90% of patients from their study that were diagnosed with recurrences in the third month also had late AFR) [38].

We found that presence of AFR in the blanking period correlates with AFR in the first year, and this is a predictor of the 6-month score for late (6 to 12 months) AFR. Therefore, we consider that electrical activity in the first 3 months should be interpreted carefully, as almost 70% of patients from our cohort continued to present AFR after the blanking period, while new cases of recurrences after the first 3 months were diagnosed in only 9.5% of patients.

We identified three independent predictors for the late AFR (“AFR duration in the first month”, “number of AFR recurrences between 1 and 3 months”, and “number of SVPB per 24 h at 6-month continuous ECG monitoring”), that can explain, independently or computed in a risk score, up to 70% of AFR between 6 and 12 months. We also found that recurrences between 6 and 12 months and their duration are the only most important independent predictors for AFR over 1 year. We support the theory that patients with AFR between 6 to 12 months differ from patients with earlier AFR or latter AFR and this period should be considered as “intermediate” [36]. In our study, 73% of patients with AFR between 6 and 12 months continued to have AFR after the first year.

Most of the AFR risk scores are based on multiple morphological and echocardiographic parameters, with the following also including very early or early AFR: MB-LATER risk score (refers to AFR in the first 2–3 months, gender, type of AF, LAD, and bundle branch blocks) with better prognostic value for patients with persistent AF [39,40], BASE-AF2 (that also includes body mass index > 28 kg/m^2^, left atrium diameter, type of AF, duration, and smoking status) in patients with paroxysmal AF for long-term outcome [41]. Bavishi et al. also found very early AFR as an independent predictor for AFR [42].

Repeat ablation is recommended for symptomatic AFR, but the timing is controversial. A second ablation is not recommended earlier than 3 months, and most studies recommend deferring for at least 3 to 6 months before reintervening [34].

Successful management with RFCA may require more than one procedure, but a good outcome relies on close monitoring, since not all symptoms can be attributed to AF recurrences. In patients with symptomatic AFR we consider 6 months is a reasonable time to decide on reintervention for patients with symptomatic AF recurrences, but not long enough to be completely sure that all patients with AFR really need a second RFCA. Based on the 6-month risk score, patients with low-risk for AFR (score < −0.474), should be deferred for reintervention the next 6 months, and irrespective of their 6-month risk score, a final decision is made at 12 months based on AF recurrences between 6 and 12 months. As AF RFCA becomes more approachable even in low-volume AF ablation centres, we suggest a 12-month follow-up with an assessment at 6 months. The 6-month intermediate risk score is designed for AF recurrences for the next 6 months but a final decision for a re-do ablation can be made after the one-year assessment.

### 4.4. Limitations

The main limitation of the current study resides in the small number of patients from a single centre with a low volume of interventions. We are fully aware of this limitation that may raise uncertainties regarding the results. Despite this limitation, our risk scores are statistically validated and show a good power of prediction. As other studies with fewer patients from the past proved their added value to clinical practice [43], we consider our findings may guide the clinical approach of paroxysmal AF patients.

Lack of continuous monitoring using an internal loop recorder (ILR) is another limitation, as several AF recurrences might have been lost, mostly if episodes were pauci- or asymptomatic.

## 5. Conclusions

The predictors for AF recurrences both at 6 and 12 months explain a high number of recurrences. Decision for reintervention may be guided by our two-step strategy with a 6-month intermediate score that proved to be a useful tool in guiding attitude. Six months is a reasonable time, but not long enough to be completely sure that all patients with AFR will still need a second intervention, while a longer follow-up to 12 months is needed for a better prediction of AFR.

## Figures and Tables

**Table 1:** Baseline characteristics of patients (*n* = 40).

Characteristics	Results
Age at ablation time, years, mean ± SD (95% CI)	56.43 ± 9.66 (53.33–59.52)
Time from AF diagnosis, years, mean ± SD (95% CI)	3.45 ± 2.34 (2.70–4.20)
**Treatment, *n* (%)**	
Propafenone	16 (40%)
Amiodarone	13 (32%)
Flecainide	4 (10%)
Betablockers	29 (72%)
Statins	16 (40%)
ACEI	25 (62%)
**Associated conditions, *n* (%)**	
Arterial hypertension	27 (67%)
Dyslipidemia	19 (47%)
Type 2 diabetes mellitus	7 (17%)
BMI (kg/m^2^), mean ± SD (95%CI)	29.83 ± 4.13 (28.51–31.15)
**Procedure characteristics, *n* (%)**	
Complete pulmonary vein isolation	32 (80%)
Cavo-tricuspid ablation	3 (7%)
**Echocardiographic parameters, mean ± SD (95% CI)**	
LVEF, %	56.00 ± 5.71 (54.17–57.83)
LV mass, g/m^2^	87.20 ± 13.24 (82.96–91.44)
LA anteroposterior diameter, mm	39.82 ± 4.44 (38.40–41.24)
LA volume, mL/m^2^	38.14 ± 8.78 (35.33–40.95)
RA diameter, mm	37.33 ± 5.19 (35.66–38.99)

ACEI = angiotensin conversion enzyme inhibitors; BMI = body mass index, LVEF = left ventricular ejection fraction; LV = left ventricle; LA = left atrium; RA = right atrium.

**Table 2:** Atrial fibrillation assessment at different intervals of time.

Characteristics	0–3 Months	3–6 Months	6–12 Months	After 12 Months
Patients with AFR, *n* (%)	19/40 (47)	11/40 (27)	11/40 (27)	11/40 (27)
AFR/patient, *n* (%)				
1 episode	5/19 (26)	3/11 (27)	3/11 (27)	3/11 (27)
2 episodes	6/19 (32)	2/11 (18)	3/11 (27)	0 (0)
3 episodes	3/19 (16)	0 (0)	0 (0)	1/11 (9)
More than 4 episodes	5/19 (26)	6/11 (55)	5/11 (45)	7/11 (64)
AFR duration/patient *				
<12 h	12/19 (63)	10/11 (91)	6/11 (54)	2/11 (18)
12–24 h	5/19 (26)	1/11 (9)	4/11 (36)	5/11 (45)
>24 h	1/19 (5)	0 (0)	1/11 (9)	4/11 (36)
Symptomatic AFR **	17/19 (89)	10/11 (91)	10/11 (91)	11/11 (100)
AFR diagnosed by self-monitoring, *n* (%)	17/19 (89)	11/11 (100)	10/11 (91)	11/11 (100)
AFR diagnosed by continuous ECG monitoring, *n* (%)	8/19 (42)	4/11 (36)	5/11 (45)	8/11 (73)

AFR = atrial fibrillation recurrence. * Duration was monitored using Holter ECG or serial ECGs in symptomatic patients. ** Symptoms referred to palpitations, dyspnea, and fatigability.

**Table 3:** Characteristics for patients with and without AFR between 6–12 months.

Characteristics	No AFR	AFR	*p*-Value
Patients, *n* (%)	29/40 (72.5)	11/40 (27.5)	0.006
Age, years, mean ± SD (95% CI)	57.48 ± 9.95 (53.70–61.27)	53.64 ± 8.65 (48.82–59.45)	0.266
Gender M, *n* (%)	19/29 (66)	10/11 (91)	0.1
AF history, years, mean ± SD (95% CI)	3 (4.3)	3 (3)	0.929
Body mass index, kg/m^2^, mean ± SD (95% CI)	29.32 ± 4.33 (27.38–30.88)	31.88 ± 3.99 (29.02–34.73)	0.138
PVI, *n* (%)			
Complete	25/29 (86.2)	7/11 (63.6)	0.11
Incomplete	4/29 (13.8)	4/11 (36.4)	0.11
Associated conditions, *n* (%)			
Arterial hypertension	19/29 (65)	8/11 (72)	0.66
Dyslipidemia	13/29 (44)	6/11 (54)	0.58
Diabetes	5/29 (17)	2/11 (18)	0.94
AFR presence, *n* (%)			
0–1 month	5/29 (17)	7/11 (64)	0.004
1–3 months	6/29 (21)	7/11 (64)	0.01
3–6 months	4/29 (14)	7/11 (64)	0.002
SVPB/24 h at ECG monitors, median (IQR)			
at 1 month	57.5 (215)	450 (1201)	0.079
at 3 months	29.5 (110)	124 (961.98)	0.005
at 6 months	36.23 (99.16)	830 (2424.34)	0.009
Treatment, *n* (%)			
Class I AAD	1/29 (3)	6/11 (54)	0.001
Class III AAD	0	2/11 (18)	0.018
Betablockers	22/29 (76)	10/11 (91)	0.28

Data are presented as mean ± SD (%), medians, and as numbers (percentages). 95% CI = 95% confidence interval of the difference. IQR: interquartile range. M = male, PVI = pulmonary vein isolation, AFR = atrial fibrillation recurrenc, SVPB = supraventricular premature beats, AAD = antiarrhythmic drugs.

**Table 4:** Characteristics for patients with and without AFR after 12 months.

Characteristics	No AFR	AFR	*p*-Value
Patients, *n* (%)	29 (72.5)	11 (27.5)	0.006
Age, years, mean ± SD (95% CI)	57.46 ±9.3 (53.70–61.22)	53.50 ±8.86 (47.16–59.84)	0.257
Gender M, *n* (%)	21/29 (72)	8/1 (73)	0.98
AF history, years, mean ± SD (95% CI)	3.4 ± 2.34 (2.45–4.35)	3.65 ± 2.76 (1.66–5.63)	0.915
Body mass index, kg/m^2^, mean ± SD (95% CI)	29.98 ± 4.24 (28.26–31.69)	31.22 ± 4.30 (28.15–34.30)	0.621
PVI, *n* (%)			
Incomplete	5/29 (17.2)	3/11 (27.3)	0.47
Complete	24/29 (82.8)	8/11 (72.7)	0.47
Associated conditions, *n* (%)			
Arterial hypertension	18/29 (62)	9/11 (82)	0.28
Dyslipidemia	13/29 (45)	6/11 (54)	0.58
Diabetes mellitus	4/29 (14)	3/11 (27)	0.31
AFR presence, *n* (%)			
0–1 month	7/29 (24)	5/11 (45)	0.18
1–3 months	8/29 (28)	5/11 (45)	0.28
3–6 months	6/29 (21)	5/11 (45)	0.11
6–12 months	3/29 (10)	8/11 (73)	0.001
SVPB/24 h at ECG monitors, median (IQR)			
at 1 month	57.5 (215)	450 (1201)	0.079
at 3 months	40 (108.5)	132 (1148)	0.068
at 6 months	38 (105.75)	277 (2275)	0.023
at 12 months	31.5 (73.78)	1290 (2288.56)	0.001
Treatment, *n* (%)			
Class I AAD	3/29 (10.3)	2/11 (18.2)	0.5
Class III AAD	0	1/11 (9.1)	0.062
Betablockers	23/29 (79.3)	9/11 (81.8)	0.85

Data are presented as mean ± SD (%), medians, and as numbers (percentages). 95% CI = 95% confidence interval of the difference. IQR: interquartile range. M = male, AF = atrial fibrillation, AFR = atrial fibrillation recurrence, PVI = pulmonary vein isolation, SVPB = supraventricular premature beats, AAD = anti-arrythmia drug.

## Data Availability

All data are kept in hospital records and are available on request.

## Appendix A

**Table A1:** Atrial fibrillation recurrences correlations.

Outcome	Independent Characteristics	r Correlation	*p*-Value
Presence of AFR (0–3 months)	Complete PVI	−0.401	0.010
	AF history from first diagnosis	0.033	0.840
	Number of AFR 0–3 months	0.939	0.001
	AFR 3–12 months	0.608	0.001
	Number of AFR 3–12 months	0.601	0.001
	AFR after 12 months	0.159	0.350
	SVPB/24 h at 1 month ECG monitoring	0.301	0.059
	SVPB/24 h at 3 months ECG monitoring	0.451	0.003
Presence of AFR (3–12 months)	Complete PVI	−0.387	0.014
	Number of AFR 3–12 months	0.969	0.001
	AF on ECG monitoring	0.542	0.001
	AFR after 12 months	0.523	0.001
	SVPB/24 h at 1 month	0.221	0.170
	SVPB/24 h at 3 months	0.615	0.001
	SVPB/24 h at 6 months	0.494	0.001
	SVPB/24 h at 12 months	0.646	0.001
Presence of AFR after 12 months	Complete PVI	−0.116	0.528
	AFR 3–12 months	0.523	0.001
	AFR 3–6 months	0.262	0.123
	AFR 6–12 months	0.585	0.001
	Number of AFR 3–6 months	0.224	0.189
	Number of AFR 6–12 months	0.568	0.001
	SVPB/24 h at 1 month	0.376	0.024
	SVPB/24 h at 3 months	0.370	0.026
	SVPB/24 h at 6 months	0.388	0.021
	SVPB /24 h at 12 months	0.609	0.001
AF on 48 h ECG monitor (0–3 months)	AF on self-monitoring 0–3 months	0.329	0.038
AF on 48 h ECG monitor (3–12 months)	AF on self-monitoring 3–12 months	0.542	0.001

AFR = atrial fibrillation recurrence; AF = atrial fibrillation; PVI = pulmonary vein isolation; SVPB = supraventricular premature beats.

**Table A2:** Selection of independent predictors by bivariate linear regression/univariate analysis for late AFR (6–12 months).

	Variables	Nagelkerke R^2^	Hosmer–Lemeshow Test	*p*-Value Regression Coefficient	AUROC	*p*-Value AUROC
1	AFR presence 0–1 month	0.244	-	0.011	0.726	0.031
2	AFR number 0–1 month	0.165	0.135	0.044	0.719	0.036
3	AFR duration 0–1 month	0.114	0.063	0.091	0.672	0.101
4	AFR 1–3 months	0.202	-	0.020	0.707	0.048
5	AFR number 1–3 months	0.298	0.564	0.009	0.746	0.019
6	AFR duration 1–3 months	0.202	0.598	0.025	0.715	0.039
7	SVPB/24 h at 3 months ECG monitor	0.270	0.407	0.059	0.769	0.012
8	AFR 3–6 months	0.291	-	0.005	0.744	0.020
9	AFR number 3–6 months	0.315	0.747	0.007	0.759	0.013
10	SVPB/24 h at 6 months ECG monitor	0.480	0.381	0.096	0.776	0.009

AFR = atrial fibrillation recurrence; SVPB = supraventricular premature beats.

**Table A3:** Multivariate models of AFR between 6–12 months.

Model Number	Model 1	Model 2	Model 3
Parameters	RN01, RD01, RN13, RN36, SVPB/24 h6 m	RN01, RD01, RN13, SVPB/24 h6 m	RD01, RN13, SVPB/24 h 6 m
Nagelkerke R^2^	0.735	0.728	0.707
Hosmer–Lemeshow Test	0.126	0.129	0.131
AIC	30.05	28.45	27.55
BIC	39.71	36.50	34.00

RN01 = number of AFR in the first month; RD01 = duration of AFR in the first month; RN13 = numbers of AFR between 1–3 months; RN36 = number of AFR between 3–6 months; SVPB/24 h6 m = supraventricular premature beats per 24 h at 6 months ECG monitor. AIC = Aikake’s Information Criterion; BIC = Bayesian Information Criterion.

**Table A4:** Multivariate Model Information: statistical characteristics of 6-month SCORE (for late AFR 6–12 months).

Multivariate Model	Variable 1 Arctan (Duration of AFR during the First Month)	Variable 2 Arctan (Number of AFR 1–3 m)	Variable 3 Loge (SVPB/24 h at 6 m Holter ECG)
VIF	1.029	1.291	1.264
Coefficient	2.255	2.081	0.752
Coefficient–Standard Error	1.159	1.042	0.342
Coefficient–Significance	*p* = 0.05	*p* = 0.04	*p* = 0.02
Intercept	6.05		
Intercept–Standard Error	2.1		
Intercept–Significance	*p* = 0.004		
AUROC	0.899 (0.782–1.0)		
Nagelkerke Pseudo-R^2^	0.591		
Hosmer–Lemeshow *p*-value	0.452		
AIC	33.135		
BIC	39.579		

AFR = atrial fibrillation recurrence; SVPB/24 h6 m = supraventricular premature beats per 24 h at 6 months continuous ECG monitoring; m = month.

**Table A5:** Selection of independent predictors by bivariate linear regression/univariate analysis for very late AFR (over 12 months).

	Variables	Nagelkerke R^2^	Hosmer–Lemeshow Test	*p*-Value Regression Coefficient	AUROC	*p*-Value AUROC
1	AFR presence 6–12 months	0.401	-	0.002	0.792	0.007
2	AFR duration 6–12 months	0.410	0.446	0.005	0.804	0.005
3	SVPB/24 hours at 3 months ECG monitor	0.215	0.440	0.078	0.704	0.066

AFR = atrial fibrillation recurrence; SVPB = supraventricular premature beats; AUROC = area under the ROC curve.

**Table A6:** Multivariate models assessment for AFR over 12 months.

Model Number	Model 1	Model 2	Model 3	Model 4	Model 5
Parameters	AFR 6–12 m	RD 6–12 m	AFR 6–12 m, SVPB/24 h3 m	RD 6–12 m, SVPB/24 h3 m	AFR 6–12 m, RD 6–12 m
Nagelkerke R^2^	0.449	0.428	0.455	0.421	0.452
Hosmer–Lemeshow Test	-	0.271	0.630	0.609	0.882
AIC	9.56	12.05	36.75	37.12	13.11
BIC	12.94	15.32	41.58	41.78	18.02

AFR 6–12 m = atrial fibrillation recurrence between 6 and 12 months, RD 6–12 months = recurrence duration between 6 and 12 months, SVPB/24 h3 m = supraventricular premature beats per 24 h at 3 months continuous ECG monitoring. AIC = Aikake’s Interpretation Criteria, BIC = Bayesian Interpretation Criteria.
